# Supplementary material for: Outcomes and Challenges in Noncommunicable Disease Care Provision in Health Facilities Supported by Primary Health Care System Strengthening Project in Sri Lanka: A Mixed-Methods Study
Source: Healthcare (Basel). 2023 Jan 9;11(2):202. doi: 10.3390/healthcare11020202 (PMC9859051; doi:10.3390/healthcare11020202)
Supplement: Supplementary file 1 [file healthcare-11-00202-s001.zip › healthcare-2042031-supplementary Annexs.pdf]

Annex S1: Proforma for extraction of data for assessing quality of care among those registered with diabetes and/or hypertension in the nine selected PMCIs supported by PSSP in Sri Lanka, 2021

Socio Demographic Details						
Date of data extraction:						
Personal Health Number:						
Name of the PMCI:						
Age (in completed years):			Date of Birth (optional):			
Gender			[ ] Male		[ ] Female	[ ] Transgender
Place of residence		[ ] Urban	[ ] Semi- Urban			[ ] Rural / Estate (plantation)
Marital status:	[ ] Single	[ ] Married	[ ] Widow	[ ] Divorced		[ ] Separated

Details of care at PMCI	
Date of registration	
Number of visits made to PMCI in last one year (from the date of data extraction)	
Date of last visit to PMCI	
Patient is availing care for	DM [ ] HTN [ ] CVD risk [ ]
Blood Pressure Measurement during last visit	Done [ ] Not Done[ ]
If Blood Pressure measured	SBP: DBP:
Date of latest blood glucose measurement	
Type of laboratory	Within PMCI [ ] Public facility within cluster [ ]<br>Private facility [ ]
If Blood Glucose measured	FBS: PPBS: RBS:
Date of latest lipid profile	
Type of laboratory	Within PMCI [ ] Public facility within cluster [ ]<br>Private facility [ ]
If lipid profile measured	TC: TG: LDL:<br>HDL:
Date of latest RFT	
Type of laboratory	Within PMCI [ ] Public facility within cluster [ ]<br>Private facility [ ]
If RFT done	Urea: Creatinine:
Date of latest Fundus examination	
Type of facility	Within PMCI [ ] Public facility within cluster [ ]<br>Private facility [ ]
Impression of fundus examination	
Date of latest ECG	
Type of facility	Within PMCI [ ] Public facility within cluster [ ]<br>Private facility [ ]

Impression of ECG	
Date of latest foot examination	
Type of facility	Within PMCI [ ] Public facility within cluster [ ]<br>Private facility [ ]
Impression of foot examination	
Visited any secondary or tertiary hospital in last one year (from the date of data extraction)	Yes [ ] No [ ]
If yes, was it referred from PMCI	Yes [ ] No [ ]
Reason for referral	
Patient referred back to PMCI	Yes [ ] No [ ]

Annex-S2: Interview guide for understanding challenges with retention in care and provision of quality NCD care as perceived by healthcare providers

How is the population under the PMCI is being screened for NCDS	Probe for type of screening activities conducted since the implementation of PSSP, whether it is camp based, or in OPDs as opportunistic screening, any other method, average attendance in each screening day, location of screening, involvement of community volunteers, religious or political members for conducting the screening activity, challenges if any
In your opinion, what is the response for such screening from activities from the public?	Probe for method of spreading the information regarding the camp to the public, involvement of volunteers or other community members for such activity, activity participation by the community, push from village leaders, any challenges or resistance for conducting or participating in the screening activity, other challenges if any.
What kind of challenges were faced while conducting the screening activities	Probe for adequate health workforce, place, time, conducive environment, equipment and other materials for screening, team cooperation, acceptance of the community for screening activities, other challenges faced if any
What kind of challenges were faced in referring, registration and treatment initiation of the patient for further services at HLC/ PMCI	Probe for mechanism of referral of patients from the outreach camps, challenges in patient reaching the PMCI from such referral centres, maintenance of screening records for registration and initiation of treatment, reason for patients dropping out before registration, treatment initiation and continuation of treatment, other challenges if any
In your opinion, what are the reason for people prefer to private sector	Probe for accessibility of private sector, difference in service if any, cleanliness, any other preferences
What kind of tests are being done to monitor the patients for complications	Probe for each test as per the guidelines, availability of the test, time delay from prescription, sample collection and availability of results for review to the physician, petty cash for small fund for maintenance services
What kind of challenges are there in monitoring the patients for complications	Probe for trained manpower, adequate laboratory facility, quality control of the equipment, ease of obtaining the results, challenges in maintenance of the device, repair service providers, other challenges such as non-availability of such lab services and referral to private sector for such services.
What are the challenges in referral of the patient to the apex hospital and back referral for continuum of care.	Probe for willingness and acceptance of patients to go to apex centre, functioning of PHN system in terms of referral services, utility of PHR, challenges for patients reaching apex hospital, preference for private sector, patient preference to come back to PMCI, challenges at PMCI in managing the registers of such referred back cases.

Annex-S3: Interview guide for understanding challenges with retention in care and provision of quality NCD care as perceived by patients

How was your experience while screening for NCDs?	Probe for type of screening place the patient attended, (camp, in OPDs as opportunistic screening, any other method) location of screening, ease of access, distance from home, ease of travel, challenges if any
In your opinion, what is the response for such screening from activities from your neighbours and friends?	Probe for the channel of information regarding the camp, involvement of volunteers or other community members for such activity, activity participation by the community, push from village leaders, any challenges or resistance for conducting or participating in the screening activity, average attendance in each screening day as observed by the patient,
What kinds of challenges were faced by you during screening?	Probe for adequate staff, adequate waiting time, time, conducive environment, staff behaviour, total time spent for screening, time taken after sample collection and availability of results,
What kind of challenges were faced after referral, registration and treatment initiation for further services at HLC/ PMCI	Probe for mechanism of referral, challenges in patient reaching the PMCI, ease of registration and receiving treatment based on screening results, coordination between screening team, registration team and the treating doctor, when PHN was issued, use of PHN, use of PHR, reason for people not registration/ initiating the treatment/ not following up for continuation of treatment, other challenges if any
In your opinion, what are the reason for people prefer to private sector	Probe for accessibility of private sector, difference in service if any, cleanliness, staff response, time difference, any other preferences
What kind of tests are being done to monitor for complications of NCDs	Probe for aware of any test for NCD, availability of the test, time delay from prescription, sample collection and availability of results for review to the physician, how frequently these tests are being done, importance of these tests
What kind of challenges are there in monitoring the patients for complications	Probe for availability of laboratory services at the PMCI and referral to private sector for such services, other challenges if any,
What are the challenges in referral to the apex hospital for further management and back referral for continuum of care.	Probe for reason for referral to apex centre, ease of reaching and receiving care at apex hospitals, use of PHN system in terms of referral services, utility of PHR, challenges for patients reaching apex hospital, preference for private sector for such referral services, preference to come back to PMCI or continuation of service at apex hospital, challenges in receiving care after coming back to PMCI, other challenges, if any.
