# Supplementary material for: Outcomes and Challenges in Noncommunicable Disease Care Provision in Health Facilities Supported by Primary Health Care System Strengthening Project in Sri Lanka: A Mixed-Methods Study
Source: Healthcare (Basel). 2023 Jan 9;11(2):202. doi: 10.3390/healthcare11020202 (PMC9859051; doi:10.3390/healthcare11020202)
Supplement: Supplementary file 1 [file healthcare-11-00202-s001.zip › healthcare-2042031-supplementary Tables.pdf]

Table S1: Prevalence of common NCD risk factors stratified by age, gender and PMCI among the individuals who underwent screening for NCD risk in the selected PMCIs supported by the PSSP in Sri Lanka from June 2019 to May 2021

**Characteristics**	**Tobacco smoking**	**Alcohol Use**	**Obese[1]**	**High Blood Pressure[2]**	**High Blood Sugar[3]**	**High Cholesterol[4]**	**>30% CVD risk[5]**
	% (95% CI)	% (95% CI)	% (95% CI)	% (95% CI)	% (95% CI)	% (95% CI)	% (95% CI)
Total	**7.8 (7.5-8.2)**	**13.3 (12.8-13.7)**	**11.7 (11.3-12.1)**	**27.4 (26.8-28.0)**	**13.6 (13.1-14.0)**	**43.1 (42.2-44.0)**	**1.7 (1.5-1.9)**
Age (in years)							
18-24	4.4 (1.6-9.4)	8.8 (4.6-14.9)	8.8 (4.6-14.8)	5.2 (2.1-10.4)	2.3 (0.5-6.5)	17.8 (10.5-27.3)	0 (0-4.4)
25-34	7.0 (5.4-9)	12.5 (10.2-15)	18.4 (15.8-21.3)	10.3 (8.2-12.6)	5.3 (3.8-7.1)	30.6 (26.4-35.1)	0.2 (0-1)
35-44	7.2 (6.5-8)	13.2 (12.3-14.2)	13.5 (12.6-14.4)	15.1 (14.2-16.1)	8.7 (8-9.5)	40.9 (39.1-42.8)	0.3 (0.2-0.5)
45-54	7.7 (7.0-8.4)	13.9 (13.0-14.8)	14.1 (13.2-15)	24.4 (23.3-25.5)	13.8 (12.9-14.7)	48.0 (46.2-49.8)	0.9 (0.7-1.2)
55-64	9.9 (9.1-10.7)	14.9 (13.9-15.9)	11.8 (10.9-12.7)	35.2 (33.9-36.5)	16.9 (15.9-18.0)	45.6 (43.8-47.5)	2 (1.6-2.5)
≥65	6.6 (5.9-7.4)	10.9 (9.9-11.8)	6.6 (5.8-7.3)	42.4 (40.9-43.9)	14.2 (13.1-15.3)	39.5 (37.4-41.6)	4.4 (3.8-5.1)
Gender							
Male	23.1 (22.1-24.2)	40.4 (39.2-41.7)	6.3 (5.7-6.9)	30.6 (29.5-31.7)	14.8 (13.9-15.7)	36.0 (34.3-37.7)	1.3 (1-1.6)
Female	0.6 (0.5-0.8)	0.7 (0.5-0.8)	14.8 (14.2-15.4)	27.6 (26.8-28.3)	13.6 (13.1-14.1)	47.0 (45.8-48.2)	1.9 (1.7-2.2)
PMCI							
PMCI 1	8.3 (6.2-10.9)	12.8 (10.1-15.9)	12.3 (9.7-15.2)	23.9 (20.5-27.6)	14.4 (11.6-17.6)	26.2 (22.5-30.1)	0.2 (0-1)
PMCI 2	11.5 (9.9-13.3)	20.8 (18.8-22.9)	7.8 (6.6-9.2)	17.3 (15.5-19.2)	15.9 (14.1-17.7)	58.9 (56.3-61.4)	0.3 (0.1-0.7)
PMCI 3	5.8 (4.6-7.2)	7.4 (6.1-8.9)	7.9 (6.6-9.3)	7.9 (6.5-9.5)	8.0 (6.8-9.4)	40.8 (38.4-43.2)	NA
PMCI 4	8.3 (7.3-9.5)	18 (16.5-19.5)	11.3 (10.1-12.6)	9.9 (8.7-11.1)	16.7 (15.3-18.3)	60.1 (55.0-65.0)	0.3 (0.1-0.6)
PMCI 5	7.6 (7.0-8.3)	11.4 (10.7-12.1)	15.5 (14.7-16.3)	32.7 (31.7-33.7)	14.7 (14.0-15.5)	31.9 (29.5-34.4)	2.6 (2.3-3)
PMCI 6	10.7 (8.3-13.7)	18.4 (15.2-21.9)	9.6 (7.3-12.4)	26.8 (23.1-30.8)	12.5 (9.6-15.9)	66.1 (60.7-71.3)	0.9 (0.3-2.2)
PMCI 7	4.2 (3.5-5)	10.2 (9.2-11.3)	10.5 (9.4-11.6)	31.4 (29.8-33.1)	13.0 (11.8-14.2)	41.2 (38.9-43.5)	1.8 (1.3-2.3)
PMCI 8	8 (7.1-9)	14.3 (13.1-15.5)	8.5 (7.6-9.5)	34.7 (33.1-36.3)	10.1 (9.1-11.2)	39.3 (37.6-40.9)	2 (1.5-2.6)
PMCI 9	15.2 (12.8-17.7)	16.1 (13.7-18.7)	10.9 (9-13.2)	34.2 (31.1-37.4)	15.4 (13.1-18)	67.8 (62.4-73)	1.1 (0.5-2)

[1]Individuals with BMI ≥ 30; [2]Individuals with systolic blood pressure of ≥140 mmHg or diastolic blood pressure of ≥90 mmHg; [3]Individuals with fasting blood sugar of ≥ 126 mg/dl or random blood sugar of ≥ 200 mg/dl; [4]Individuals with total cholesterol of ≥ 200 mg/dl; [5]CVD risk deduced from WHO chart and documented in the participant register maintained at PMCI

Abbreviation: PMCI- Primary Medical Care Institution; NCD- Non-communicable diseases; PSSP- Primary healthcare System Strengthening Project

Table S2: Demographic and clinical characteristics of individuals with diabetes and/or hypertension registered for NCD care in the nine selected PMCIs supported by the PSSP of Sri Lanka, June 2021

Characteristics	Only DM, n (%)	DM with HTN, n (%)	Only HTN, n (%)	Total, n (%)
**Total, N (%)[1]**	**217 (32.1)**	**132 (19.6)**	**326 (48.3)**	**675 (100)**
Age (Mean ± SD)	55.4 ± 11.0	59.9 ± 10.3	60.3 ± 11.3	58.6 ± 11.2
**Gender**				
Male	58 (26.7)	40 (30.3)	95 (29.1)	193 (28.6)
Female	159 (72.3)	92 (69.7)	231 (70.9)	472 (71.4)
**Residence**				
Urban	5 (2.3)	11 (8.3)	9 (2.8)	25 (3.7)
Semi-urban	16 (7.4)	19 (14.4)	57 (17.5)	92 (13.6)
Rural/Estate	196 (90.3)	102 (77.3)	260 (79.8)	558 (82.7)
**Marital status**				
Single	4 (1.8)	1 (0.8)	3 (0.9)	8 (1.2)
Married	142 (65.4)	102 (77.3)	218 (66.9)	462 (68.4)
Widow	10 (4.6)	2 (1.5)	12 (3.7)	24 (3.6)
**PMCI**				
PMCI 4	12 (5.5)	10 (7.6)	42 (12.9)	64 (9.5)
PMCI 2	44 (20.3)	12 (9.1)	26 (8.0)	82 (12.2)
PMCI 3	25 (11.5)	7 (5.3)	25 (7.7)	57 (8.4)
PMCI 6	18 (8.3)	20 (15.2)	52 (16.0)	90 (13.3)
PMCI 8	45 (20.7)	18 (13.6)	54 (16.6)	117 (17.3)
PMCI 9	27 (12.4)	5 (3.8)	24 (7.4)	56 (8.3)
PMCI 7	15 (6.9)	15 (11.4)	51 (15.6)	81 (12.0)
PMCI 5	17 (7.8)	29 (22.0)	34 (10.4)	80 (11.9)
PMCI 1	14 (6.5)	16 (12.1)	18 (5.5)	48 (7.1)
**Duration since registration[2]**				

≤ Three months	19 (8.8)	2 (1.5)	23 (7.1)	44 (6.5)
Three to six months	16 (7.4)	7 (5.3)	14 (4.3)	37 (5.5)
Six to Twelve months	47 (21.7)	29 (22.0)	64 (19.6)	140 (20.7)
≥ Twelve months	135 (62.2)	94 (71.2)	225 (69.0)	454 (67.3)
**Median (IQR) number of PMCI visit in last one year[3]**	6 (4-9)	6 (4-8)	6 (4-9)	6 (4-9)

[1]Row percentage with total number of individuals included in the study as denominator; [2]In reference to date of data extraction; [3]the median number of visits are calculated only among those with ≥ twelve months of duration since registration;

Abbreviations: PMCI- Primary Medical Care Institution; NCD- Non-communicable diseases; PSSP- Primary healthcare System Strengthening Project; DM- Diabetes Mellitus; HTN- Hypertension
